# Exon Level Transcriptomic Profiling of HIV-1-Infected CD4^+^ T Cells Reveals Virus-Induced Genes and Host Environment Favorable for Viral Replication

**DOI:** 10.1371/journal.ppat.1002861

**Published:** 2012-08-02

**Authors:** Michaël Imbeault, Katia Giguère, Michel Ouellet, Michel J. Tremblay

**Affiliations:** 1 Centre de Recherche en Infectiologie, Centre Hospitalier Universitaire de Québec - CHUL, Faculté de Médecine, Université Laval, Québec City, Québec, Canada; 2 Département de Microbiologie-Infectiologie et Immunologie, Faculté de Médecine, Université Laval, Québec City, Québec, Canada; NIH/NIAID, United States of America

## Abstract

HIV-1 is extremely specialized since, even amongst CD4^+^ T lymphocytes (its major natural reservoir in peripheral blood), the virus productively infects only a small proportion of cells under an activated state. As the percentage of HIV-1-infected cells is very low, most studies have so far failed to capture the precise transcriptomic profile at the whole-genome scale of cells highly susceptible to virus infection. Using Affymetrix Exon array technology and a reporter virus allowing the magnetic isolation of HIV-1-infected cells, we describe the host cell factors most favorable for virus establishment and replication along with an overview of virus-induced changes in host gene expression occurring exclusively in target cells productively infected with HIV-1. We also establish that within a population of activated CD4^+^ T cells, HIV-1 has no detectable effect on the transcriptome of uninfected bystander cells at early time points following infection. The data gathered in this study provides unique insights into the biology of HIV-1-infected CD4^+^ T cells and identifies genes thought to play a determinant role in the interplay between the virus and its host. Furthermore, it provides the first catalogue of alternative splicing events found in primary human CD4^+^ T cells productively infected with HIV-1.

## Author Summary

Some previous studies have monitored HIV-1-induced gene expression in various host cell targets and tissues but the discrimination between productively infected cells and uninfected bystander cells represents a technical challenge yet to be solved. Consequently, data interpretation has always been biased towards the transcriptional response of a majority of uninfected bystander cells that were exposed to soluble factors released by virus-infected cells. Following the design of a unique and innovative molecular tool to identify cells productively infected with HIV-1 and the description of an efficient magnetic beads-based technique to separate them from uninfected bystander cells, we undertake this challenge and perform the first comparative whole-genome transcriptomic and large-scale proteomic profiling of both HIV-1-infected and uninfected bystander CD4^+^ T cells. We demonstrate herein that HIV-1- infected and uninfected bystander cells display distinctive transcriptomic signatures which might permit to identify new susceptibility and resistance factors.

## Introduction

CD4^+^ T cells – the primary cellular target of HIV-1 – are progressively depleted over the course of infection. This long-term process culminates in the onset of AIDS, a condition in which the immune system is too weak to efficiently mount an effective defence against opportunistic pathogens. Yet, HIV-1 uses only 15 proteins to disable the natural immune defences and harness the host cell machinery to complete its replicative cycle. To do so, viral proteins interact with multiple cellular proteins, perturbing the normal flow of cellular processes. Moreover, the virus influence extends beyond the cells it infects. Indeed, the apoptosis rate of uninfected bystander CD4^+^ T cells is elevated in individuals carrying HIV-1 [1]. The dichotomy between uninfected bystander and HIV-1-infected CD4^+^ T cells is an important topic to study, as a deeper understanding of HIV-1 pathogenesis mechanisms might lead to new therapeutic approaches.

Powerful technologies developed in recent years have provided high-throughput tools to study cellular dynamics. Among them, microarrays allow for the quantification of expression levels of thousands of genes at once. Since the inception of this technology, few studies have used microarrays to characterize the effect of HIV-1 on various cell types that compose the immune system. However, productive infection rates in primary human cells such as CD4^+^ T lymphocytes are very low. As microarray technology captures the average transcriptomic profile of a cell population, achieving a high level of purification of subpopulations of interest is crucial to accurately quantify any possible virus-mediated changes in the host transcriptome [2]. We recently developed a new reporter virus system that allows the efficient separation of HIV-1-infected cells from their uninfected bystander counterpart *in vitro* [3]. In the current work, we used this unique and versatile tool to define the HIV-1-induced modulation of host gene expression in one of its most worrisome cellular reservoir. We hereby provide the first time-course comparative and comprehensive study of the effect of HIV-1 in productively infected versus uninfected bystander primary human CD4^+^ T cells using Affymetrix Human Exon arrays. We pinpoint understudied genes which seemingly play important roles in the subset of CD4^+^ T cells preferentially infected by HIV-1 and provide an overview of splicing events found in this subpopulation.

## Results

**Figure 1:**
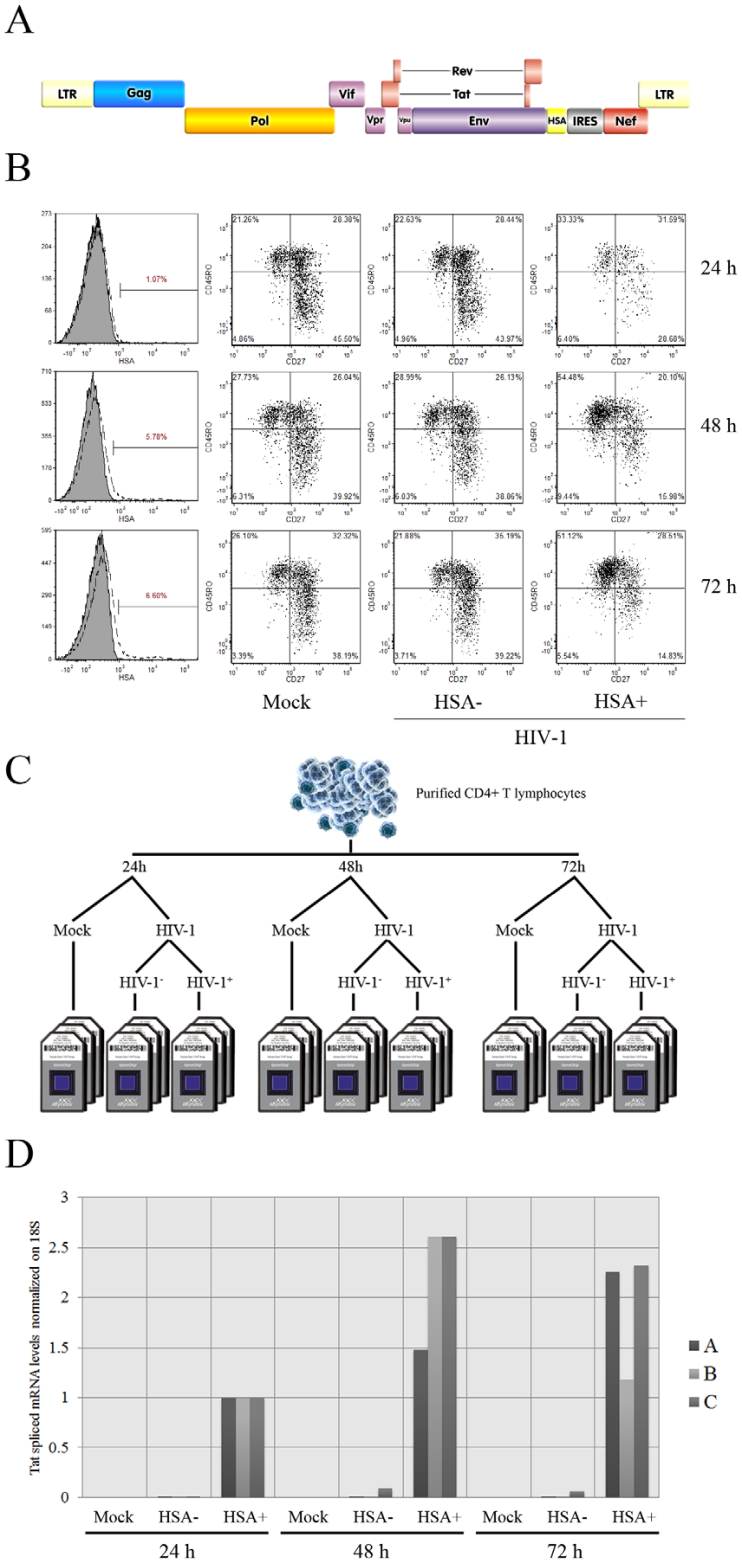
Isolation and characterization of HIV-1-infected cells. A) Design of the reporter virus used in this study. The murine reporter HSA gene ensures a rapid detection and isolation of cells productively infected with HIV-1. In this infectious molecular clone of HIV-1, the early viral gene *Nef* is expressed via an IRES sequence. The reporter virus thus expresses all viral genes and is fully replication competent. B) Flow cytometry analysis of the virus-infected cell populations. Left column: Percentage of HIV-1-infected primary human CD4^+^ T cells at 24, 48 and 72 h post-infection, as revealed by HSA staining. Second, third and fourth columns: characterization of T cell phenotypes via CD45RO and CD27 staining for mock-infected, uninfected bystander (HSA^−^) and virus-infected cells (HSA^+^). C. Experimental design for the transcriptome analysis. Purified CD4^+^ T cells (98% CD3^+^CD4^+^; data not shown) from the peripheral blood of three different donors were treated with PHA/rhIL-2 for two days, then either mock-infected or inoculated with NL4-3 BAL-IRES-HSA virus. Next, uninfected bystander cells (HSA^−^) and HIV-1-infected (HSA^+^) cell populations were separated through the use of magnetic beads. At least 200 ng of RNA were obtained from each cell population and processed for hybridization on microarrays. D) Purity levels attained after separation of the studied cell populations. qRT-PCR directed against spliced Tat mRNA was carried out to assess the presence of HIV-1-infected cells in each fraction (donors A, B and C). Results are expressed relatively to virus-infected cell fractions, as the signal in negative controls is usually undetectable. Each value is normalized by the value of 18S ribosomal RNA. All HSA-positive cell fractions are highly enriched in virus-infected cells, whereas mock-infected and uninfected bystander cell populations contain virtually no detectable traces of viral transcripts.

While an impressive amount of data has been gathered about HIV-1 and its putative relationships with various components of the immune system, the most favorable cellular microenvironment for HIV-1 establishment and replication within CD4^+^ T lymphocytes have never been fully elucidated. It is known that this retrovirus preferentially infects activated CD4^+^ T cells [4], but infection rates are very low even in this susceptible population. We confirmed this phenomenon using a replication competent reporter virus system containing all viral genes along with the small murine heat stable antigen (HSA) protein [3] (Figure 1A), which is rapidly expressed on the surface of virus-infected cells. PHA/IL-2-treated primary human CD4^+^ T cells exposed to this recombinant reporter virus reached 1.1, 5.8 and 6.6% of HSA-positive cells after 24, 48 and 72 h post-infection, respectively (Figure 1B, left panels). Although it is difficult to conclude that the enhanced percentage of HIV-1-infected CD4^+^ T cells during the first three days is strictly due to additional infection events and not due, at least partially, to cell death in cells not productively infected with HIV-1, it can be postulated that factors other than cellular activation are essential for the successful establishment of productive HIV-1 infection.

Primary human activated CD4^+^ T cells can be classified in distinct subsets which include naïve (T_naïve_/CD45RO^−^CD27^+^), intermediate memory (T_IM_/CD45RO^+^CD27^+^) and effector memory (T_EM_/CD45RO^+^CD27^−^) [5], [6]. We characterized the profile of CD4^+^ T cells most permissive to productive HIV-1 infection by staining for CD45RO, CD27 and HSA. Results show that HIV-1 displays a preference for T_EM_ cells that is increasing over time (Figure 1B, compare columns showing mock, HSA^−^ and HSA^+^) with enrichment ratios of 1.5, 1.9 and 2.3 in HIV-1-infected versus uninfected bystander cells at 24, 48 and 72 h post-infection. However, the phenotype does not fully account for HIV-1 selectivity as we find a significant amount of virus-infected T_naïve_ and T_IM_ cells, even though these subsets are less susceptible to productive HIV-1 infection compared to T_EM_ cells (enrichment ratios of 0.7, 0.4 and 0.4 for T_naïve_ and 1.1, 0.8 and 0.8 for T_IM_).

### HIV-1-infected CD4^+^ T cells display a distinct transcriptomic profile

We next performed a comparative microarray analysis of mock-infected, uninfected bystander (HSA^−^) and HIV-1-infected primary human CD4^+^ T cells (HSA^+^) in an attempt to identify both virus-induced genes and intracellular environment most permissive to productive infection. To this end we magnetically separated virus-infected cells from three different donors on the basis of HSA expression from the whole cell population exposed to the R5-tropic reporter virus for 24, 48 or 72 h (Figure 1C). We next confirmed productive HIV-1 infection in isolated cell fractions using a spliced Tat-specific qRT-PCR based on the idea that such a sensitive technique allows the quantification of early expressed viral transcripts without detection of input viral RNA. Data shows a strong signal in the fractions containing HSA-expressing cells (called HIV Pos), while uninfected bystander cell fractions (called HIV Neg) and mock-infected (called Mock) displayed an almost complete absence of spliced Tat, thus indicating a very high degree of cell purification (Figure 1D).

**Figure 2:**
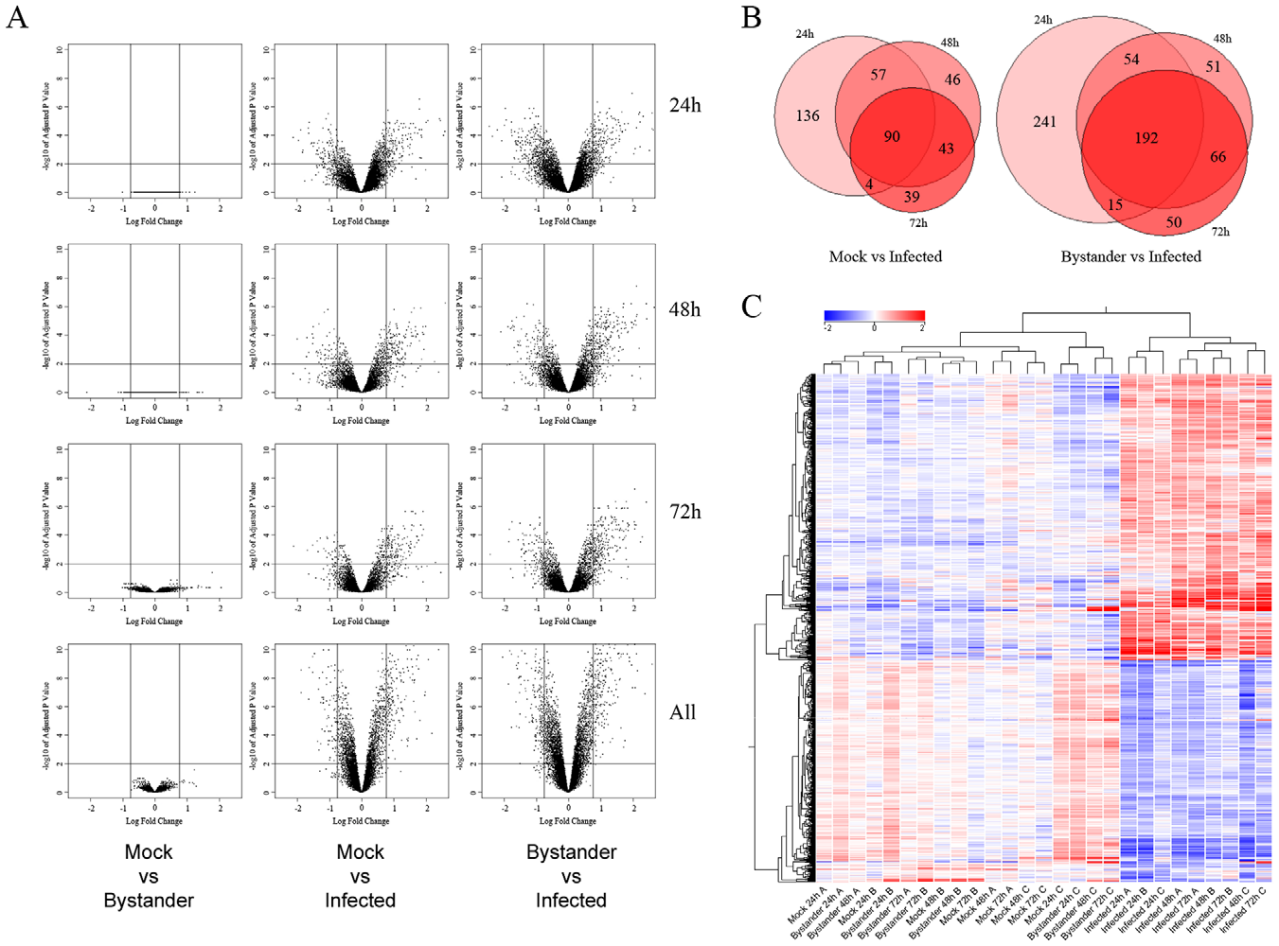
Transcriptomic profile of mock-infected, uninfected bystander and HIV-1-infected cells. A) Volcano plots from the limma analysis of the transcriptome of cells fractions described in Figure 1D, at 1% FDR and 1.7 fold-change cut-off (represented by lines). The log10 of the p-value adjusted for multiple comparisons (Benjamini and Hochberg) is used for the Y axis and the log2 of the fold change is used for the X axis. Left columns: comparison between mock-infected versus uninfected bystander cell populations shows no statistically significant genes passing both thresholds at any time point or in aggregate analysis (labelled “all”). Center columns: comparison between mock-infected and virus-infected cells. Right columns: comparison between uninfected bystander and HIV-1-infected cells. B) Proportional Venn diagrams of above data, illustrating the overlap of DEGs at 24, 48 and 72 h for mock vs infected and bystander vs infected comparisons. C) Unsupervised hierarchical clustering (per sample and per gene) of differentially-expressed genes in the dataset. The similarity between mock-infected and uninfected bystander cells is obvious. Moreover, HIV-1-infected cell samples cluster together at the 24 h time point, but diverge on a per-sample basis at 48 and 72 h, illustrating that, although a common subset of T cells are infected early on, the precise context preferred by the virus can evolve differently over time on a per-sample basis.

Thereafter, we extracted total RNA from the studied cell fractions and performed transcriptome profiling using Affymetrix Human Exon arrays, which interrogate over 5 million probes spanning all exons in the human genome. A careful analysis of differentially expressed genes (DEGs) using a false discovery rate of 1% and a cut-off of 1.7 fold showed no effect of HIV-1 on the transcriptome of uninfected bystander cell population compared with mock-infected cells at any studied time point or in aggregate (Figure 2A, left panels). Comparison between mock-infected and HIV-1-infected cells revealed 287, 236 and 176 DEGs at 24, 48 and 72 h post-infection, respectively (Figure 2A, middle panels) while the aggregate comparison of all time points yielded 289 genes. Given that no DEG was identified in the uninfected bystander cell population, we then compared cells productively infected with HIV-1 to uninfected bystander cells to improve our statistical power, the rationale being that the separation procedure allows to isolate the small percentage of virus-infected cells by removing them from the uninfected bystander cell fraction (constituting the majority of cells), creating a better signal differential than the one obtained comparing with the mock-infected fraction. By doing so, we obtained 502, 366 and 323 DEGs (at 24, 48 and 72 h) in HIV-1-infected cells while the aggregate comparison yielded 464 DEGs (Figure 2A, right panels). Proportional Venn diagrams depict the relative distribution of DEGs and their evolution through time for these two comparisons (Figure 2B). An overview of all modulated genes is presented in Figure 2C as a hierarchical cluster. It can be concluded that most DEGs identified in HIV-1-infected target cells (Figure 2C) are stable at least during the studied period, whereas expression of a small cluster of genes increases significantly over time. Complete lists of DEGs that were found to be modulated are depicted in Dataset S1.

### The transcriptomic profile of HIV-1-infected cells is consistent with that of highly activated effector T cells

**Figure 3:**
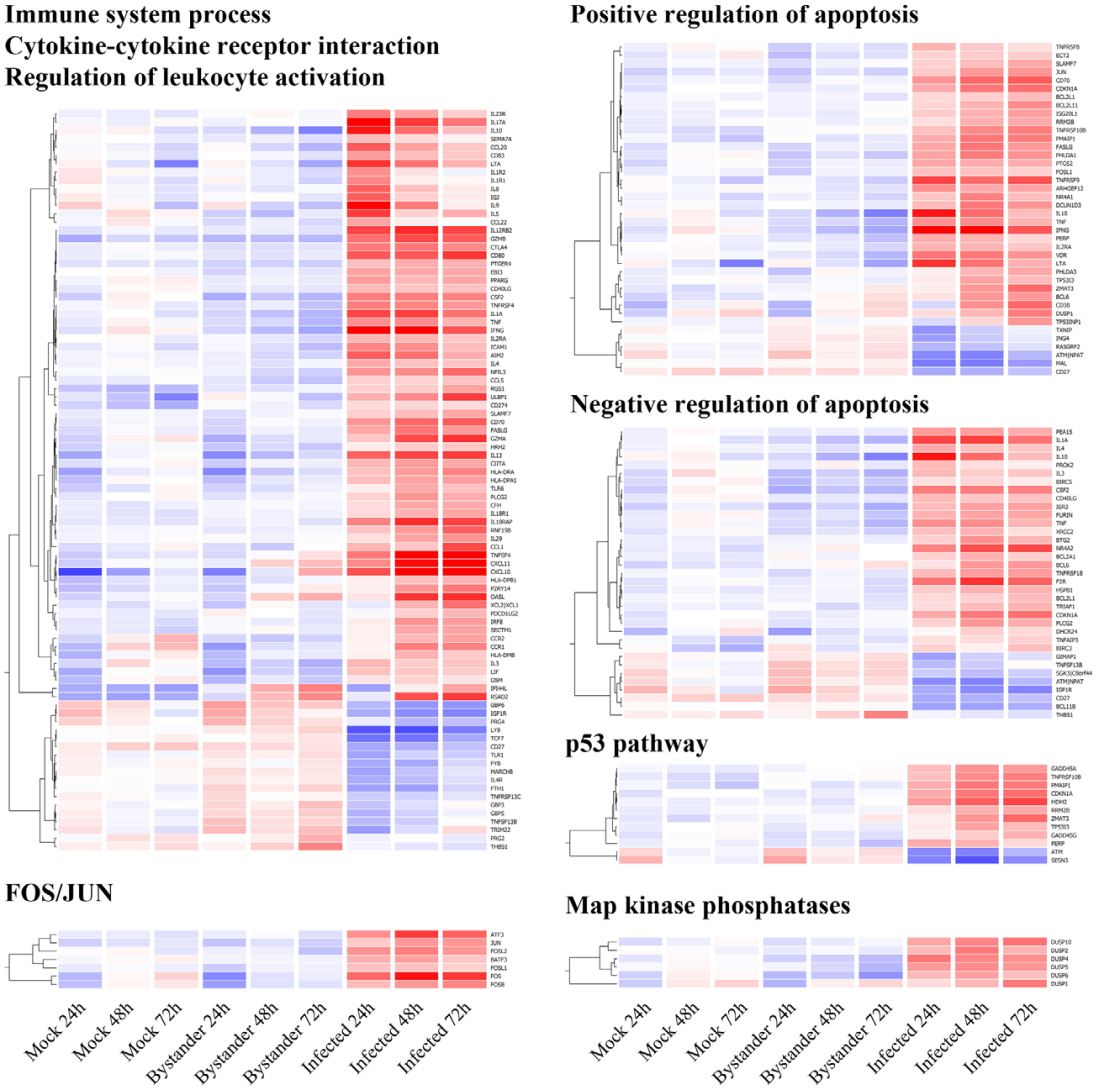
Gene Ontology overrepresentation analysis of genes differentially expressed in HIV-1-infected cells. Unsupervised hierarchical clustering (per gene) of statistically over-represented Gene Ontology & KEGG pathway categories according to DAVID analysis. Since the first three categories (Immune system process – p = 1.9×10^−17^, Cytokine-cytokine receptor interaction – p 1×10^−29^, Regulation of leukocyte activation – p = 1.4×10^−9^) overlap significantly, they are presented as a single cluster. Positive and negative regulation of apoptosis (p = 5.8×10^−7^ and 6×10^−6^, respectively) are both part of the Regulation of apoptosis category, but are here presented separately to better illustrate the absence of trend, in either direction. FOS/JUN (p = 2.3×10^−5^), the p53 pathway (p = 2.2×10^−6^) and Map kinase phosphatase (p = 2.3×10^−5^) are also identified as being over-represented and have implication for HIV-1 biology. All p-values mentioned above are corrected for multiple testing according to Bonferroni. The same color scale as Figure 2 applies.

We broadly defined the characteristics of the dataset using the metadata clustering engine DAVID, which identifies enriched biological themes within a list of genes using various biological annotation sources [7]. Using the combined list of 835 DEGs identified in all comparisons performed, we found the following statistically over-represented categories: immune system process, cytokine-receptor interaction, regulation of leukocyte activation, Map kinase phosphatases, FOS/JUN related genes, positive/negative regulation of apoptosis and p53 pathways (Figure 3).

The first three identified categories overlap significantly and contain markers for Th1 (i.e. IFN-γ, TNF-α, TNF-β, IL-1A, IL-3, and TBX21/T-bet), Th2 (i.e. IL-4, IL-5, IL-10, and IL-13) and Th17 profiles (i.e. IL-17A, IL-17F, IL-21, IL-22, IL-23R, and IRF4), all of which are over-expressed in primary human CD4^+^ T cells productively infected with HIV-1 (Figure 3, top left). The expression patterns associated with these different profiles suggest that Th1 and Th17 cells are slightly more susceptible to infection with the studied R5-tropic reporter virus, as virus-infected cells express more IFN-γ and IL-17A than other cytokines (Figure S1). Other molecules associated with the effector phenotype and activated T cells in general are also differentially expressed (i.e. IL4R, IL18R, MCSF, GMCSF, ICAM-1, OX40, OX40L, CD27, and its counterpart CD70, CD80, CTLA4, CD69, CD40LG, IL2RA, FASLG, IL12RB2, IL18R1, IL18RAP, and IL-9). Their expression pattern is consistent with highly activated T_EM_ cells being preferentially infected by HIV-1. Several Map kinase phosphatases involved in T-cell activation are also modulated in virus-infected cells (Figure 3, bottom right).

### AP-1 plays a central role in cell permissiveness to HIV-1 infection

Fos and Jun bind together to form the AP-1 transcription factor [8], which is essential for the differentiation and proliferation of lymphocytes. Multiple AP-1 binding sites are found in the HIV-1 LTR [9] and it has been demonstrated that this transcription factor promotes viral transcription [10]. The DAVID analysis pinpointed a cluster of over-expressed Fos-related genes (i.e. Fos, Jun and related genes BATF3, ATF3, FOSB, FOSL1, and FOSL2) in virus-infected cells (Figure 3, bottom left). Interestingly, a promoter analysis of the genes modulated in HIV-1-infected cells reveals that 57% of DEGs identified in this study and 70% of core genes (differentially expressed at 3 time points) contain at least one binding site for AP-1 (Figure S2), confirming that this transcription factor is one of the core determinants of the cellular environment that is most favorable to productive HIV-1 infection in primary human CD4^+^ T cells.

### p53-dependent apoptosis is a dominant theme in HIV-1 infection

Among DEGs found precisely in HIV-1-expressing cells are genes involved in both positive and negative regulation of apoptosis (Figure 3, top right). Most of these genes appear to be an integral part of the genetic programme engaged in virus-infected cells and are stable through time. However, expression a subset of genes increases rapidly at 48 and 72 h post-infection, thus suggesting that such DEGs are a consequence rather than the determinant of viral gene expression. Further analysis shows that these genes are implicated in p53-dependent apoptosis – specifically CDKN1A/p21, MDM2, GADD45A, GADD45G, TNFRSF10B (TRAIL), ATM, ZMAT3, PMAIP1 (NOXA), BCL2L11, PERP, TP53I3, RRM2B, and SESN3 (Figure 3, bottom right). Additionally, the p53 pathway is identified by the DAVID analysis as the most significantly over-represented ontology category among the 180 genes exclusive to the 48 h and 72 h time points (p<0.0001; data not shown). The expression pattern of these genes over time is in agreement with the numerous reports of p53-related apoptosis in HIV-1-infected cells following DNA damage caused by the virus integration process [11], [12], [13], [14], [15]. However, patterns of differential expression of p53-regulating genes emerge as early as 24 h, before HIV-1-induced apoptosis occurs. For example, MDM2, a factor responsible for the degradation of p53 [16], is over-expressed in virus-infected cells. On the other hand, ATM, a gene involved in phosphorylation and inactivation of MDM2 [17], is under-expressed during this same period. This suggests that cells with low potential for p53 activation are more susceptible to productive HIV-1 infection, perhaps due to their slower reaction time for triggering p53-dependent apoptosis following viral integration, giving the virus more time to replicate actively.

### Bibliography analysis provides an intuitive overview of the complex dataset

**Figure 4:**
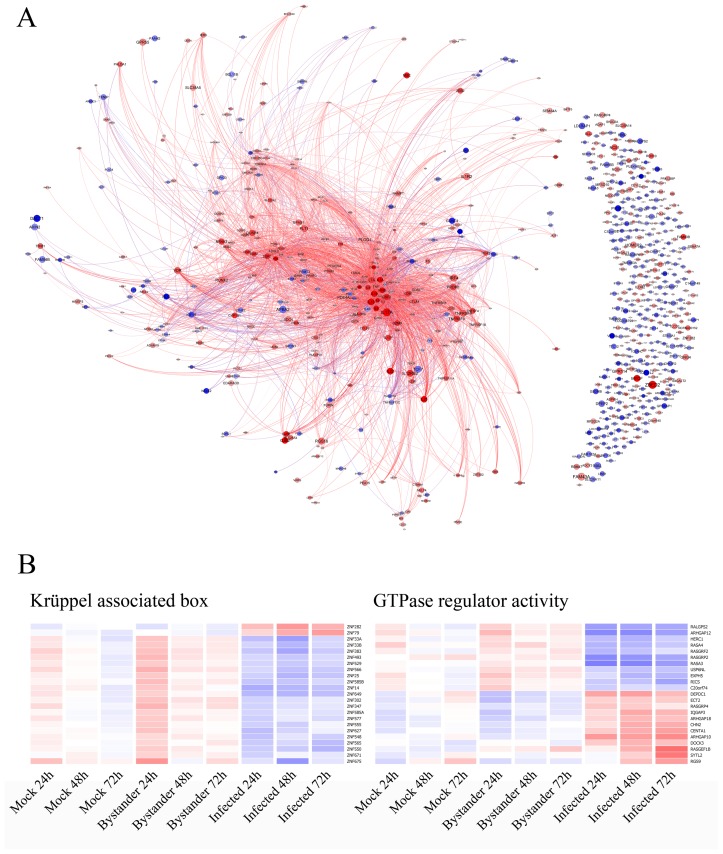
Bibliography analysis and focus on neglected genes. A) Co-citation analysis was performed with Bibliosphere and exported to Gephi. The data shown is from the HIV-1-infected versus uninfected bystander cells comparison at the 24 h time point. Pairs of genes with at least three abstracts co-citing the genes at the sentence level were considered as significant. Red genes are over-expressed in virus-infected cells while blue genes are under-expressed. The size of circles is inversely correlated to the corrected p-value obtained in the experiment (i.e. the bigger the circle, the smaller the p-value). The length of links between nodes is inversely correlated with the number of abstracts found in Pubmed for the two related genes. Gephi allows dynamic filtering and interaction with the data. Readers are encouraged to download Gephi (http://gephi.org) and use the graph file (Dataset S2) to explore the dataset dynamically. Poorly studied genes having no significant relationship with other members are shown in the right portion of the graph. B) Hierarchical clustering of gene categories (Kruppel associated box – p = 3.7×10^−3^ and GTPase regulator activity – p = 8.3×10^−4^) identified as being statistically over-represented among understudied genes.

The DAVID analysis is useful for broad categorization but is ultimately insufficient to fully extract biological significance from the dataset. A bibliography analysis was thus performed to visualize known relationships between genes present in the dataset. Bibliosphere (Genomatix) extracts relationships from co-citation of gene names (including synonyms) in abstracts from Medline. The resulting network illustrated in Figure 4A is consistent with the previous analysis, as the already identified over-represented categories cluster together. Moreover, the analysis uncovered numerous DEGs closely related to these clusters, but missed by the DAVID analysis. For example, it pinpointed CDC25A, CDC25C, and CDC14A near the p53 genes cluster. The pattern of expression of these genes is consistent with cells being most permissive to HIV-1 infection when entering in the M-G2 phase of the cell cycle [18]. The graph can also help identify small clusters of genes that would otherwise have gone unnoticed. Notably, furin and PCSK5 proteases, which are both known to participate in the cleavage of viral glycoprotein gp160 [19], [20], [21], [22], show opposite expression patterns. Indeed, furin is over-expressed in virus-infected cells, while PCSK5 is under-expressed at the 24 h time point. This implies that, although both proteases can cleave the viral envelope *in vitro*, furin is favored in acute HIV-1 infection studies. The graph file is available as Dataset S2 and can be explored dynamically by using graph visualization software Gephi (http://gephi.org/).

### The analysis of a cluster of understudied genes reveals the importance of KRAB-ZNFs and GTPase regulators in HIV-1 and lymphocyte biology

It was recently suggested that poorly characterized genes deserve a more careful analysis [23]. We performed a careful literature analysis and found 178 unconnected genes for which no known relationship with other genes in the dataset is currently described and/or little information is available (Figure 4A). We repeated the DAVID analysis on this subset of genes and found two over-represented protein domains, i.e. Krüppel associated box (KRAB) and GTPase regulator activity (Figure 4B).

The KRAB domain is mainly found in KRAB-ZNFs, a large family of mammalian transcription factors responsible for negative regulation of transcription through chromatin remodelling via their association with KAP1 [24]. They have been recently implicated in control of endogeneous retroelements [25]. Most of the modulated KRAB-ZFPs are under-expressed in virus-infected cells, which might indicate that their absence creates a permissive environment for HIV-1, probably by playing a role in T-cell regulation. Two of the identified KRAB-ZFPs can modulate crucial components of HIV-1 and lymphocyte biology. Indeed, overexpression of ZNF383 inhibits the transcriptional activities of AP-1 [26], while ZNF675 can suppress TRAF6-induced activation of NF-κB and c-Jun N-terminal kinase [27]. Interestingly, the two members that are over-expressed in virus-infected cells show mutations in the MLE motif of their KRAB domain (i.e. ZNF79 and ZNF282) (Figure S3), a pattern associated with loss of repression potential for KRAB-ZFPs [28]. It should be noted that ZNF79 is also a known p53 target [29].

The second over-represented category contains GTPase regulators, mainly of the RHO/RAC family. These influence a variety of cellular processes such as endocytic trafficking, actin dynamics and cell growth by affecting the rate of GTP hydrolysis by GTPases [30]. Many of the identified members either participate in T-cell signaling responses or directly influence proteins known to be important for HIV-1 entry in human primary CD4^+^ T cells, such as HERC1 (under-expressed in virus-infected cells) that is known to regulate ARF6 [31], [32].

### Candidate restriction and permissive factors for HIV-1 are found amongst poorly studied genes

Amongst the list of DEGs in HIV-1-infected cells, most genes showing high levels of differential expression are poorly studied. Since we isolated cells productively infected with HIV-1 from the total population (primarily composed of uninfected bystander cells), under-expressed genes potentially impede HIV-1 at some step of its replicative cycle, while the reverse holds true for over-expressed genes. For example, TRIM22, known for its role in inhibition of HIV-1 transcription [33], is under-expressed in virus-infected cells. Of note TRIM22 RNA levels progressively return to normal, suggesting that HIV-1 can overcome its effects over time (Table 1). Transcription factors known to repress HIV-1 transcription such as YY1 [34] and BCL11B/CTIP2 [35] are also found to be under-expressed in virus-infected cells. MARCH8, a regulator of vesicular transport of proteins between cellular compartments, was recently identified in a large scale siRNA screen as a top candidate for the inhibition of HIV-1 infection [36] and is found under-expressed in virus-infected cells in the present dataset.

**Table 1:** qRT-PCR confirmation of genes of interest identified in the dataset.

		24 h			48 h			72 h			Pearson correlation (excluding Mock)
		Mock	Bystander	Infected	Mock	Bystander	Infected	Mock	Bystander	Infected	
**ZBED2**	Microarray	1.00	0.86	3.28	1.00	0.74	3.03	1.00	0.73	2.86	0.87
	PCR	1.00	0.78	1.68	1.00	0.74	2.16	1.00	1.13	3.53	
**MYOF**	Microarray	1.00	0.87	3.77	1.00	0.71	2.74	1.00	0.66	2.06	0.94
	PCR	1.00	1.20	2.14	1.00	0.82	2.60	1.00	0.63	1.69	
**GJB2**	Microarray	1.00	1.03	4.78	1.00	0.65	3.59	1.00	0.92	3.38	0.83
	PCR	1.00	0.79	2.45	1.00	0.92	6.36	1.00	1.23	2.41	
**GJB6**	Microarray	1.00	1.03	3.29	1.00	0.82	3.21	1.00	0.96	2.62	0.79
	PCR	1.00	1.32	1.59	1.00	1.24	3.80	1.00	0.91	2.32	
**TRIM22**	Microarray	1.00	1.20	0.50	1.00	1.30	0.88	1.00	1.77	1.50	0.87
	PCR	1.00	1.95	0.24	1.00	1.11	0.60	1.00	1.40	0.93	
**CLC**	Microarray	1.00	1.52	0.35	1.00	1.90	0.36	1.00	2.41	0.29	0.95
	PCR	1.00	3.01	0.08	1.00	1.09	0.14	1.00	2.41	0.14	
**DFNA5**	Microarray	1.00	1.01	1.55	1.00	0.96	2.60	1.00	1.02	3.11	0.99
	PCR	1.00	0.90	2.06	1.00	1.14	8.11	1.00	0.85	8.80	

Expression levels of ZBED2, MYOF, GJB2, GJB6, TRIM22, CLC and DFNA5 were confirmed by qRT-PCR. The Pearson correlation coefficients indicate that the level of similarity between qRT-PCR quantification and microarrays is very high for all studied candidates.

We hereby present a list of the most promising understudied genes in regard to their importance for HIV-1 pathobiology and T lymphocyte biology. For example the transcription factor ZBED2 is highly over-expressed in HIV-1-infected cells. Although little is known about its function, this gene has been reported to be over-expressed in differentiated T cells [37], [38]. Its precise direct and indirect roles in the regulation of HIV-1 expression or lymphocyte differentiation remain to be more clearly defined. GJB2 and GJB6 are connexins that are members of the gap junction protein family involved in the formation of cell-cell channels [39]. It is possible that they play a role in viral entry or cell-cell transmission. GSDMB and DFNA5 are two members of the gasdermin protein family – DFNA5 has been recently shown to participate in p53-dependent apoptosis [40]. MYOF is a membrane-associated protein involved in both caveolin and clathrin-mediated endocytosis pathways along with membrane resealing after damage [41]. This gene is highly over-expressed in HIV-1-infected cells and could participate in the repair of the plasma membrane following the budding of a multitude of virions, allowing the cell to live longer and to produce more viruses. The expression pattern of CLC (also called galectin-10) differs from that of other modulated genes. CLC mRNA levels are significantly diminished in virus-infected cells whereas they steadily increase over time in uninfected bystander cells (although not reaching statistical significance). CLC has been shown to be a crucial determinant of Treg suppressor function [42]. PCR quantification for the aforementioned genes reveals excellent concordance with the microarray data (Table 1). We are currently investigating the precise role of some of these genes in the HIV-1 infection process and T-cell biology.

### Catalogue of alternative splicing events found in virus-infected cells further define the cellular environment most permissive for HIV-1 infection

**Figure 5:**
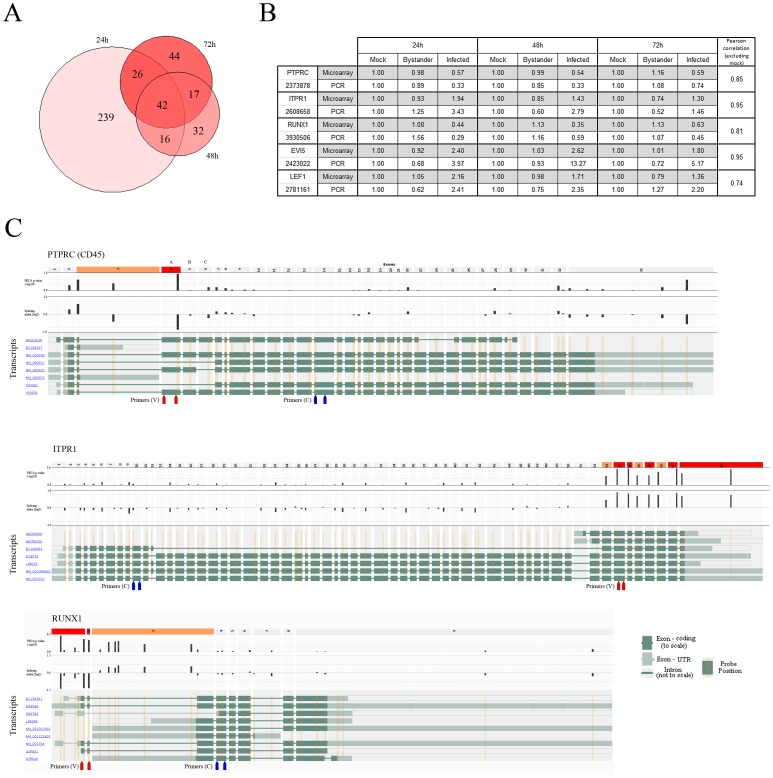
Alternative splicing events are found in HIV-1-infected cells. A) Proportional Venn diagram of the number of splicing events identified in virus-infected cells versus uninfected bystander cell populations at 24, 48 and 72 h post-infection. B) Confirmation by qRT-PCR of splicing events of interest. Pearson correlation coefficients indicate that the level of similarity between qRT-PCR quantification and microarrays is very high for all studied candidates. C) Overlay of the PECA-SI algorithm p-values and the splicing index (from aggregate comparisons) for each probeset on Affymetrix Exon arrays on a representation of selected genes in their genomic context. The exons that pass both filters (p-value and fold change – dotted line on their respective plots) are coloured red and those that pass only one filter colored orange. Red and blue arrow pairs represent primer sets used for qRT-PCR confirmation of splicing events (see above). PCR primers for confirmation of splicing events are also displayed (red – variable region between isoforms, blue – constant region). Top panel: PTPRC, better known as CD45, was selected as a target to validate our approach and filtering strategy, as CD45RO memory cells are more prone to be productively infected by HIV-1. Exon 4 is the one associated with the RA variant predominant in naïve cells. Middle panel: ITPR1, which plays a role in lymphocyte activation. Bottom panel: RUNX1 (AML1/EVI1), which is a master regulator of hematopoietic development important for T cell differentiation [46] and is known to have multiple isoforms arising from alternative promoter usage [47].

Affymetrix Exon arrays allow for the detection of alternative splicing events. Using the PECA-SI algorithm [43], we detected 323, 129 and 107 differential splicing events in transcripts of virus-infected versus uninfected bystander CD4^+^ T cells at 24, 48 and 72 h post-infection, respectively (Figure 5A and Dataset S3; see Methods for filtering parameters). Similarly to gene expression, more events were detected when comparing HIV-1-infected to uninfected bystander cells than to mock-infected ones, and no alternative splicing events were detected in the uninfected bystander population.

We looked for a splicing event known to occur in HIV-1-infected cells. In T lymphocytes, CD45 isoforms are a marker of naive (CD45RA) and memory phenotypes (CD45RO) – the latter being preferentially infected by HIV-1 [44]. This is indeed reflected in the data, as exon 4 of CD45 (corresponding to CD45RA) is under-expressed in virus-infected cells, implying that this fraction contains more CD45RO (Figure 5C, top).

We next focused our attention on events susceptible to be important in the biology of both HIV-1-infected and CD4^+^ T cells. Multiple events are detected in the C-terminal portion of inositol 1,4,5-triphosphate receptor (ITPR1), yielding a short isoform containing only the calcium-channel domains of the protein (Figure 5C, center). ITPR1 plays a role in lymphocyte activation [45] and this isoform could represent a constitutionally active form of the receptor present in highly activated and/or memory cells. RUNX1 (AML1/EVI1) is a master regulator of hematopoietic development important in T-cell differentiation [46] and is known to have multiple isoforms arising from alternative promoter usage [47] (Figure 5C, bottom). We identify a short isoform of RUNX1 being enriched specifically in virus-infected cells. The isoforms of RUNX1 have been implicated in different stages of haematopoiesis [47]. However their precise role in T cells or potential interaction with HIV-1 are still unclear. Other interesting alternative splicing events include an isoform of LEF1, a transcription factor implicated in the regulation of HIV-1 transcription [48], and EVI5, an oncogene implicated in cell cycle control [49] – both are RUNX1 interaction partners. qRT-PCR confirmation for all the aforementioned events was performed and results are highly consistent with microarray-acquired data (Figure 5B).

## Discussion

In this manuscript, we provide the first comparative analysis of exon-level transcriptomic profiles between HIV-1-infected primary human CD4^+^ T cells and their uninfected bystander cell counterparts. By doing so, we define the virus-induced genes and microenvironment most favorable to allow productive HIV-1 infection and show that even within a population of activated CD4^+^ T cells, the permissive environment for HIV-1 infection is very specific. The profile of virus-infected cells is consistent with activated/effector memory CD4^+^ T cells expressing high levels of cytokines. We found that Th1 and Th17 were to some extent more permissive to virus infection in this specific *in vitro* experimental setting. The expression patterns of most genes identified in this study are in agreement with the current literature in regard to HIV-1 and lymphocyte activation status, which provide significance to our observations. However, it must be emphasized while we rediscovered many well-studied genes and pathways known to be important for HIV-1, the precise function of a large number of other genes that were identified in this work is still currently poorly described. We believe that efforts should be made to understand their function, as yet unexplored avenues might allow deepening our understanding of the interplay between HIV-1 and lymphocyte biology.

The design of our microarray experiment captures both the transcriptomic portrait of highly permissive cells and the changes induced by the virus itself. While a clear discrimination between theses two events is complicated by confounding factors such as asynchronous infection and a potential cell death of some CD4^+^ T cells, a longitudinal analysis of the data allows us to achieve a comparison of gene expression patterns between HIV-1-infected and uninfected bystander cells. We concluded that genes for which levels change significantly over time in virus-infected cells are directly modulated by HIV-1. As an example, the p53 apoptosis pathway is clearly induced in virus-infected cells at 48 and 72 h post-infection. However, the vast majority of differentially expressed genes are relatively stable over time in virus-infected CD4^+^ T cells and thus define the transcriptomic programme of a subpopulation preferentially infected by HIV-1. In this context, over-expressed genes are potential viral permissiveness factors, while underexpressed genes are candidate restriction factors. Functional assays are currently underway to determine which of the newly identified candidate genes play a key role in HIV-1 and/or lymphocyte biology.

One of our primary objectives was to identify changes occurring in uninfected bystander CD4^+^ T cells exposed to HIV-1, as previous studies have reported dramatic effects in cells exposed to virions or its components. To this end, we purposely left the initial viral inoculum in our purified CD4^+^ T cell populations to allow for putative virus-mediated signal transduction events. We were surprised to find no significant changes in the transcriptome of the uninfected bystander cell population at least at early time points after HIV-1 infection (i.e. 24, 48 and 72 h). It is possible that the presence of cells other than CD4^+^ T lymphocytes is required to mediate changes in gene expression in uninfected bystander cells. We nonetheless detected an increase of apoptosis in uninfected bystander cells at late time points (data not shown). This could imply that gene regulation is not necessary for apoptosis induction. For example, direct caspase activation induced by FAS or TRAIL ligation could explain this phenomenon. It should also be noted that we used an R5-tropic variant of HIV-1, as this tropism is more representative of early infection events. However, it cannot be excluded that an X4-tropic virus would have an effect on the transcriptome of uninfected bystander cells, as this variant characteristic of late-stage infection is known to have higher apoptosis-inducing activity [50]. On a similar note, a somewhat different HIV-1-mediated gene expression pattern might be obtained when using a distinct R5-tropic virus. Additional experiments are needed to solve these issues.

As we demonstrated in this work, the isolation of human primary CD4^+^ T cells productively infected with HIV-1 is a powerful approach which amplifies the power of transcriptome analysis. We believe that further dissection of virus-infected CD4^+^ T cell subtypes could yield even more information, as the profile we obtained is undoubtedly an average of different types of susceptible subpopulations. While observations made in this manuscript describe the relationship between HIV-1 and CD4^+^ T cells, it is in the absence of the multitude of other factors influencing dynamics of infection *in vivo*. Therefore, transcriptomic analysis of virus-infected cells in more complex experimental settings such as total peripheral blood mononuclear cells or humanized mice models would provide additional insight in the intricate relationship between the virus and its host environment. We hope that the data provided here can serve as a roadmap to focus efforts on neglected aspects of T-cell and HIV-1 biology, leading to a better understanding of the complex relationship between the virus and its host.

## Materials and Methods

### Ethics statement and cells

Human peripheral blood mononuclear cells were obtained from healthy blood donors, in accordance with the guidelines of the Bioethics Committee of the Centre Hospitalier de l'Université Laval Research Center, by density-gradient centrifugation on Ficoll-Hypaque (Wisent, St-Bruno, QC). All blood donors were informed and agreed to a written consent prior to blood donation. Cells were plated in 75-cm2 flasks at 15×10^6^/mL for 2 h. The non-adherent cells from the supernatant were enriched in CD4^+^ T cells with the human CD4^+^ T Cell Enrichment Kit (Stemcell Technologies Inc., Vancouver, BC). The purity obtained was routinely higher than 98% of CD3^+^CD4^+^ T cells. Cells were cultured at 2×10^6^/ml in RPMI-1640 medium (Invitrogen, Burlington, ON) supplemented with 10% foetal bovine serum (Invitrogen), L-glutamine (2 mM) (Wisent), penicillin G (100 U/ml), streptomycin (100 µg/ml) (Wisent) and primocine (Amaxa Biosystems, Gaithersburg, MD). Cells were rested for 24 h after isolation and treated with phytohemagglutinin-L (PHA) (1 µg/ml) (Sigma-Aldrich, St-Louis, MO) and recombinant human IL-2 (rhIL-2) (30 U/ml) (AIDS Research and Reference Reagent Program, Germantown, MD) for 2 days at 37°C under a 5% CO_2_ atmosphere prior to HIV-1 infection and rhIL-2 was refreshed when the medium was changed. Human embryonic kidney 293T cells were maintained in DMEM (Invitrogen) supplemented with 10% FBS, L-glutamine, penicillin G and streptomycin.

### Virus stocks and infection

NL4-3 BAL-IRES-HSA virions were described previously [3] and produced in 293T cells using a commercial calcium phosphate kit (CalPhos Mammalian Transfection kit, Clontech, Palo Alto, CA). Cell-free supernatants were ultracentrifuged to eliminate free p24. Finally, samples were aliquoted before storage at −85°C. A homemade ELISA test was used to normalize the p24 content in all viral preparations [51]. Virus infection was achieved by inoculating primary human CD4^+^ T cells with a fixed amount of reporter virus standardized in term of p24 (i.e. 10 ng of p24 per 1×10^5^ target cells). All virus preparations underwent a single freeze-thaw cycle before initiation of infection studies. A control consisting of mock-infected cells was obtained by transient transfection of 293T cells with an equimolar amount of pCDNA 3.1 (control empty vector). Next, purified CD4^+^ T cells were treated with a volume of supernatant from 293T cells transfected with pCDNA 3.1 similar to the one used for HIV-1 infection experiments. A total of 100×10^6^ primary human CD4^+^ T cells were exposed to NL4-3 BAL-IRES-HSA and 30×10^6^ target cells were used for mock-infected controls for each of the three donors tested. In order to get about 5×10^5^ CD4^+^ T cells productively infected with HIV-1 (i.e. HSA^+^), a total of 50, 30 and 20×10^6^ cells were used to isolate an average of 5×10^5^ virus-infected cells at 24, 48 and 72 h post-infection, respectively.

### Separation of HIV-1-infected and uninfected bystander cells

Following infection of primary human CD4^+^ T cells with the fully competent HSA-encoding virions, HIV-1-infected and uninfected bystander cell populations were isolated using a previously described protocol with slight modifications [3]. In brief, all magnets were pre-cooled for one hour at 4°C. Only the first HSA-negative fraction was used to obtain uninfected bystander cells because subsequent negative fractions have an increased risk of containing HIV-1-infected cells (unpublished data). Mock-infected cells were subjected to the same procedure as the uninfected bystander fraction.

### RNA isolation

A protocol was designed for RNA isolation, as neither Trizol nor silica-based column methods could yield sufficient amounts of high quality RNA from HIV-1-infected fractions. Indeed, the positive fractions contain magnetic beads which were found to interfere with the Trizol reagent (See Protocol S1).

### Flow cytometry

Flow cytometry analyses were performed with 5×10^5^ cells that were incubated with 100 µl of wash buffer (PBS [pH 7.4], BSA 1%, and EDTA 2 mM) containing a saturating amount of a monoclonal rat anti-mouse HSA antibody (clone M1/69, PE-coupled, BD Biosciences, Mississauga, ON), anti-CD4, anti-CD3, anti-CD27, anti-CD45RO (all from BD Biosciences) or a corresponding isotype-matched control antibody for 30 min at 4°C. Cells were then washed, fixed with 2% paraformaldehyde for 30 min at 4°C and analyzed on a cytofluorometer (FACSCanto, BD Biosciences). Further analyses were performed using FCS Express V3.0 software (De Novo Software, Los Angeles, CA).

### Microarray and bioinformatics analysis

A total input of 200 ng of RNA was used to prepare targets for array hybridization using the Ambion WT Expression Kit (Applied Biosystems, Austin, TX). Data was normalized using RMA at the gene and exon level with Affymetrix Power Tools – core-level probe definition were used in both cases for results presented in this manuscript. Bioconductor package limma [52] was used to find modulated genes – an FDR of 1% and a fold change of 1.7 were used to filter the lists. A minimum signal filter of 100 on the average of three replicates was also applied. Time-wise and aggregate comparisons were done between infected, bystander and mock treated cells.

DAVID analysis was done using version 6.7 with standard parameters using the following categories: GOTERM_BP_FAT, GOTERM_MF_FAT, KEGG_PATHWAY, BIOCARTA, SP_PIR_KEYWORDS, UP_SEQ_FEATURE, SMART, INTERPRO, UCSC_TFBS. Bonferroni corrected p-values<0.001 were considered as significant.

Bibliosphere analysis was performed using version 7.24 with the compiled list of 835 modulated genes identified by limma. We settled on the presence of three co-citations or more in the same sentence in a PubMed abstract as a relationship criterion. We exported the data to Gephi (http://gephi.org/), a powerful and interactive network visualization and exploration platform for further analysis using the Gefx library. Nodes were arranged using a directed force algorithm (Force2), colored according to fold change between uninfected bystander and HIV-1-infected cells at 24 h and sized according to the –log10 of the p-value for the same comparison – these can be dynamically changed at will using the original Gephi file containing the graph and all associated data, available as Dataset S2.

Alternative splicing analysis was performed with PECA-SI. The following filters were applied: a threshold of splicing index of at least 1.7 fold, a significant DABG signal (p<0.001) in at least 3 groups (equivalent to 9 chips), probes contained in exonic regions and an FDR of 1%. Data was dynamically overlaid and visualized on both Annmap (http://annmap.picr.man.ac.uk/) via Bioconductor xmapcore and xmapbridge packages and Splice Center [53].

qRT-PCR. qRT-PCR against the Tat-spliced isoform was performed to quantify the level of enrichment of HIV-1-infected CD4^+^ T cells achieved in the HSA-positive fraction and their absence in the HSA-negative fractions. TaqMan RNA-to-CT 1-Step Kits from Invitrogen was used for quantification. The following probe and primers were used in our study: probe, TATCAAAGCAACCCACCTCC, forward primer, GAAGCATCCAGGAAGTCAGC, reverse primer, CTATTCCTTCGGGCCTGTC. PCR was performed under standard TaqMan cycling conditions using a Rotor-gene 3000 (Corbett Life Science, San Francisco, USA). SYBR Green detection was used for all subsequent PCR targets. qRT-PCR confirmation of genes was performed with the Power SYBR Green RNA-to-CT 1-Step kit from Invitrogen. Expression values were normalized to 18S and quantification was performed using the CT method. Primers for gene expression and alternative splicing confirmations are shown in Table S1.

## Supporting Information

Dataset S1 List of genes differentially expressed in all comparisons.
(XLS)

Dataset S2 Gephi graph file of the complete Bibliography analysis. The network can be dynamically filtered and modified.
(ZIP)

Dataset S3 List of alternative splicing events found in all comparisons.
(XLS)

Figure S1 Selected profile of Th related genes. Although cytokines related to Th1, Th2 and Th17 are overexpressed in virus-infected cells, Th1 related IFNG and Th17 related IL17A show higher values, hinting at a slight preference of HIV-1 for those functional subtypes.
(TIF)

Figure S2 A) DAVID overrepresentation analysis of transcription factor binding sites in the promoter region of core genes (differentially expressed at all time points), showing AP-1 as the core determinant of the observed expression pattern. The transcription factor binds 69.5% of the promoters. B) Same analysis with all DEGs. AP-1 is still significantly overrepresented, binding 57.1% of the promoters.
(JPG)

Figure S3 A) Similarity tree of KRAB-ZFPs identified in this study, according to ClustalW alignment of protein sequences. It can be noted that the overexpressed ZNF282 is a clear outlier. B) Focus on the KRAB domain of the alignment. The underlined MLE motif is severely disrupted in both the overexpressed genes ZNF282 and ZNF79, as well as ZNF393. This motif is important in the repression potential of KRAB-ZFPs.
(JPG)

Protocol S1 RNA isolation.
(DOC)

Table S1 Primers used for qRT-PCR confirmations of expression and alternative splicing.
(XLS)
