# Supplementary figures and images for: Exon Level Transcriptomic Profiling of HIV-1-Infected CD4+ T Cells Reveals Virus-Induced Genes and Host Environment Favorable for Viral Replication
Source: PLoS Pathog. 2012 Aug 2;8(8):e1002861. doi: 10.1371/journal.ppat.1002861 (PMC3410884; doi:10.1371/journal.ppat.1002861)

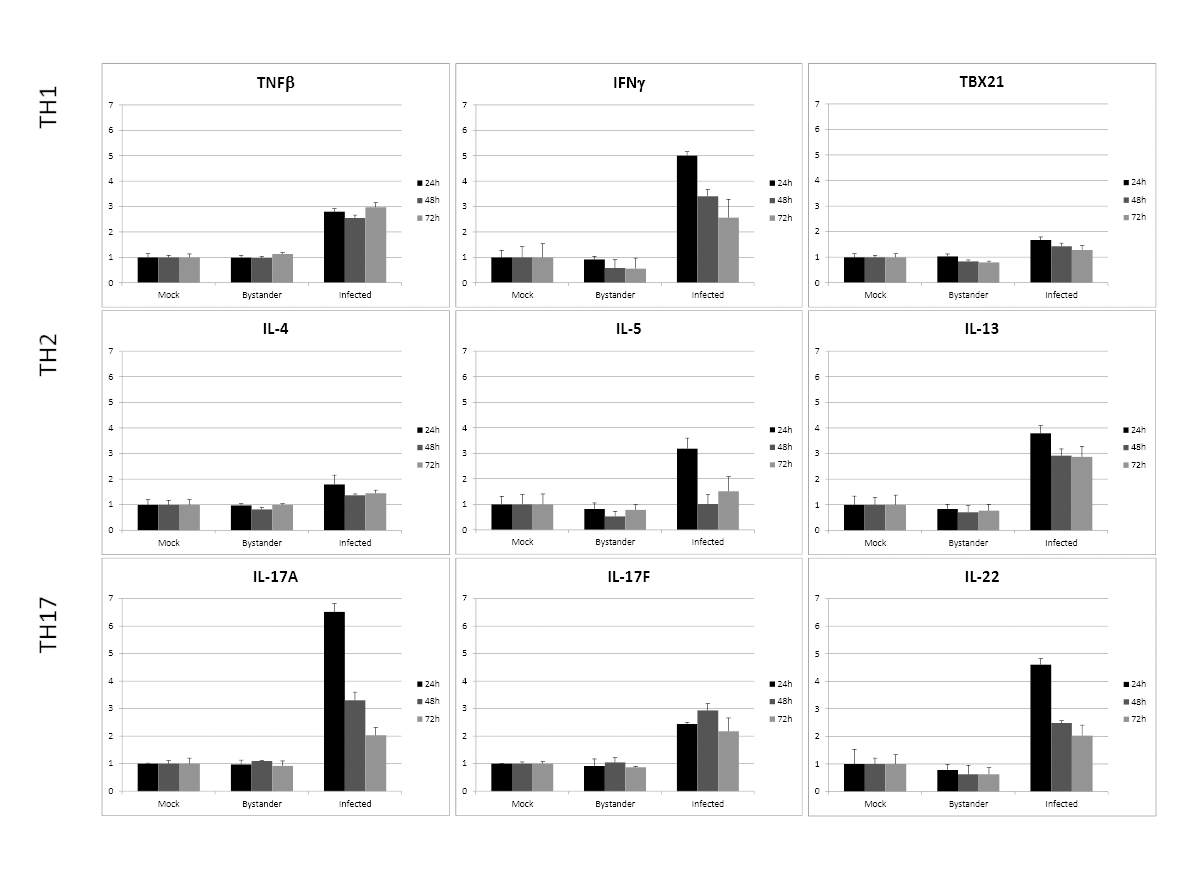

Supplement: Figure S1 — Selected profile of Th related genes. Although cytokines related to Th1, Th2 and Th17 are overexpressed in virus-infected cells, Th1 related IFNG and Th17 related IL17A show higher values, hinting at a slight preference of HIV-1 for those functional subtypes. (TIF) [file ppat.1002861.s004.tif]

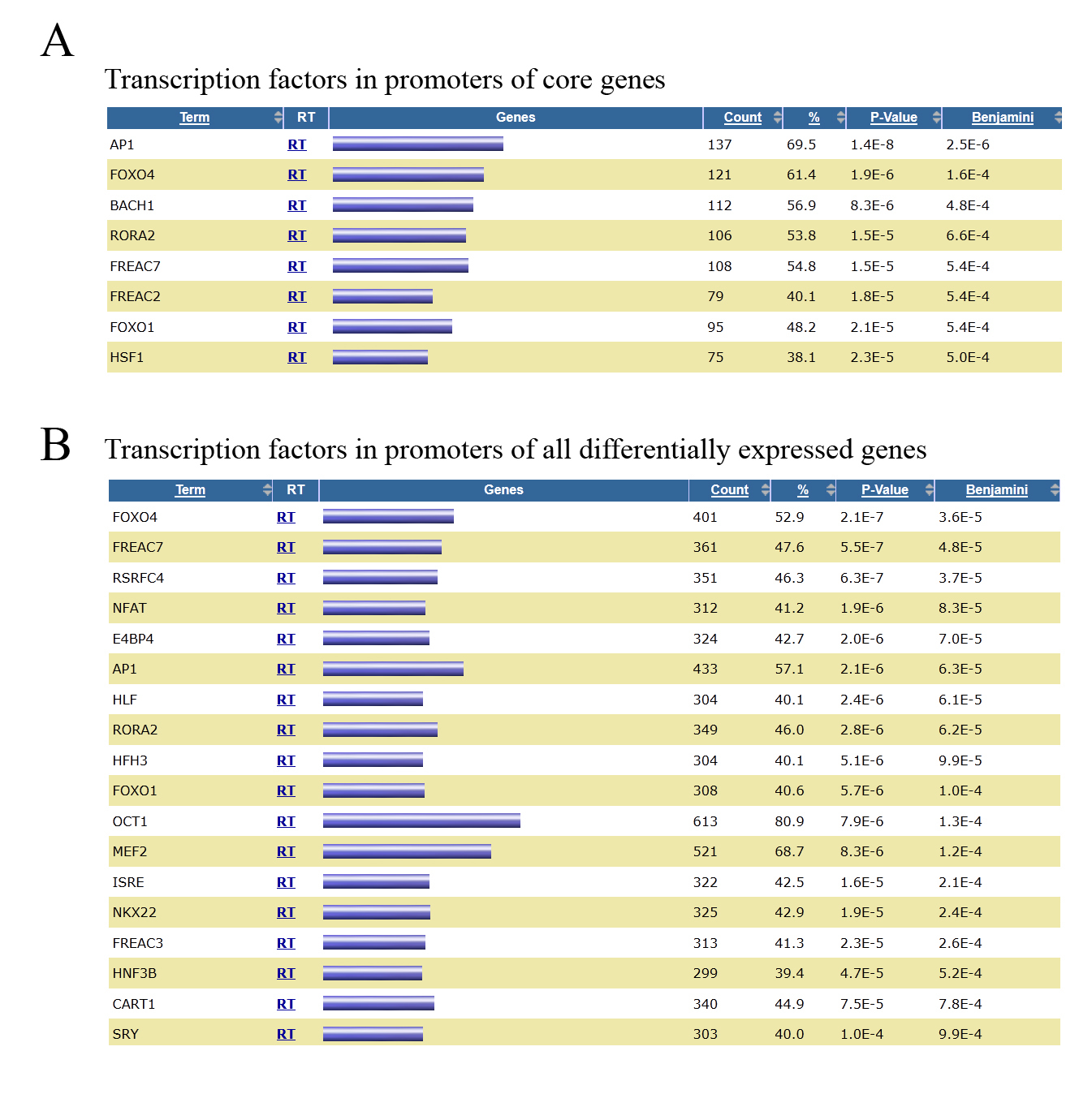

Supplement: Figure S2 — A) DAVID overrepresentation analysis of transcription factor binding sites in the promoter region of core genes (differentially expressed at all time points), showing AP-1 as the core determinant of the observed expression pattern. The transcription factor binds 69.5% of the promoters. B) Same analysis with all DEGs. AP-1 is still significantly overrepresented, binding 57.1% of the promoters. (JPG) [file ppat.1002861.s005.jpg]

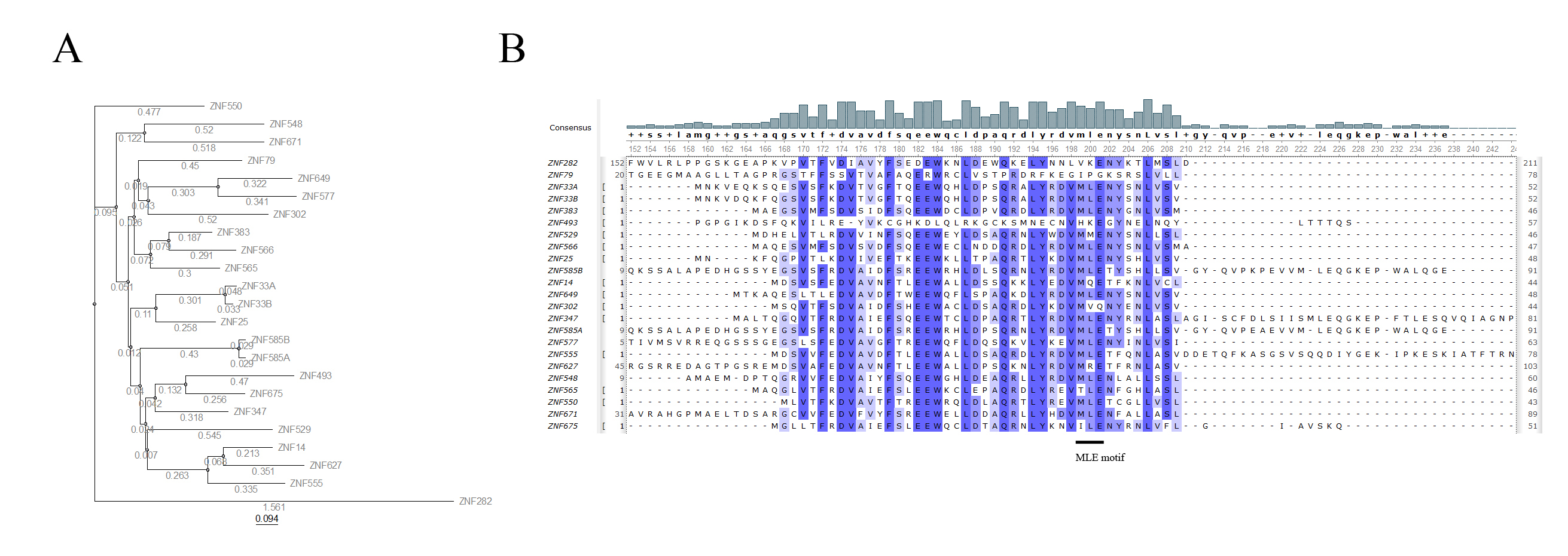

Supplement: Figure S3 — A) Similarity tree of KRAB-ZFPs identified in this study, according to ClustalW alignment of protein sequences. It can be noted that the overexpressed ZNF282 is a clear outlier. B) Focus on the KRAB domain of the alignment. The underlined MLE motif is severely disrupted in both the overexpressed genes ZNF282 and ZNF79, as well as ZNF393. This motif is important in the repression potential of KRAB-ZFPs. (JPG) [file ppat.1002861.s006.jpg]
