# Supplementary material for: Exon Level Transcriptomic Profiling of HIV-1-Infected CD4+ T Cells Reveals Virus-Induced Genes and Host Environment Favorable for Viral Replication
Source: PLoS Pathog. 2012 Aug 2;8(8):e1002861. doi: 10.1371/journal.ppat.1002861 (PMC3410884; doi:10.1371/journal.ppat.1002861)
Supplement: Protocol S1 — RNA isolation. (DOC) [file ppat.1002861.s007.doc]

**RNA isolation**

Briefly, PBS-washed cell pellets frozen at -80^o^C were submerged in 1 mL of Trizol, vortexed for 1 min and left to rest for 5 min. Thereafter, bromo-chloro-propane (BCP) (0.1 mL) was added and the mixture was vortexed for 2 min. DEPC-treated water (170 μL) was added to facilitate phase separation and the lysate was mixed by vigorous pipetting and incubated for 5 min at room temperature. Phase Lock Heavy tubes were prepared by centrifugation at 12,000g for 2 min at room temperature. The Trizol lysate was added to the Phase Lock tubes and centrifuged for 10 min at 4^o^C until phase separation. The aqueous phase was transferred to a new tube and mixed with 100% molecular-grade ethanol (400 μL). An aliquot of 550µL was transferred to a Qiagen RNeasy micro kit column and allowed to run through by centrifugation. The rest of the sample was then processed in the same column. From there, the protocol from Qiagen was followed with slight modifications. Briefly, RW1 wash was performed in two steps of 350 µL and RNA eluted with 12 µL of water. A final volume of 0.5 µL was used to quantify concentration by Nanodrop and RNA quality was assessed by Bioanalyzer nano chips (Agilent).
